# Predictive identification and design of potent inhibitors targeting resistance-inducing candidate genes from *E. coli* whole-genome sequences

**DOI:** 10.3389/fbinf.2024.1411935

**Published:** 2024-07-26

**Authors:** Abdullahi Tunde Aborode, Neeraj Kumar, Christopher Busayo Olowosoke, Tope Abraham Ibisanmi, Islamiyyah Ayoade, Haruna Isiyaku Umar, Abdullahi Temitope Jamiu, Basit Bolarinwa, Zainab Olapade, Abidemi Ruth Idowu, Ibrahim O. Adelakun, Isreal Ayobami Onifade, Benjamin Akangbe, Modesta Abacheng, Odion O. Ikhimiukor, Aeshah A. Awaji, Ridwan Olamilekan Adesola

**Affiliations:** ^1^ Department of Chemistry, Mississippi State University, Starkville, MS, United States; ^2^ Department of Pharmaceutical Chemistry, Bhupal Nobles’ College of Pharmacy, Udaipur, Rajasthan, India; ^3^ Department of Biotechnology, Federal University of Technology, Akure, Nigeria; ^4^ Institute of Bioinformatics and Molecular Therapeutics, Osogbo, Nigeria; ^5^ Department of Microbiology, Federal University of Technology, Akure, Nigeria; ^6^ Computer-Aided Therapeutic Discovery and Design Platform, Federal University of Technology, Akure, Nigeria; ^7^ Department of Biochemistry, Federal University of Technology, Akure, Nigeria; ^8^ Department of Microbiology and Biochemistry, University of the Free State, Bloemfontein, South Africa; ^9^ College of Medicine, Richmond Gabriel University, Richmond, Saint Vincent and the Grenadines; ^10^ Department of Biology, Lamar University, Lamar, TX, United States; ^11^ Department of Microbiology and Immunology, University of Louisville, Louisville, KY, United States; ^12^ Department of Chemistry, State University of New York Albany, Albany, NY, United States; ^13^ Department of Biological Sciences, University of Albany, Albany, NY, United States; ^14^ Department of Epidemiology, School of Public Health, Georgia State University, Atlanta, GA, United States; ^15^ School of Public Health, Georgia State University, Atlanta, GA, United States; ^16^ Department of Biological Sciences, University at Albany, State University of New York, Albany, NY, United States; ^17^ Department of Biology, Faculty of Science, University College of Taymaa, University of Tabuk, Tabuk, Saudi Arabia; ^18^ Department of Veterinary Medicine, Faculty of Veterinary Medicine, University of Ibadan, Ibadan, Nigeria

**Keywords:** *E. coli*, resistance, antimicrobial, inhibitors, genes, whole-genome sequences

## Abstract

**Introduction:** This work utilizes predictive modeling in drug discovery to unravel potential candidate genes from *Escherichia coli* that are implicated in antimicrobial resistance; we subsequently target the gidB, MacB, and KatG genes with some compounds from plants with reported antibacterial potentials.

**Method:** The resistance genes and plasmids were identified from 10 whole-genome sequence datasets of *E. coli*; forty two plant compounds were selected, and their 3D structures were retrieved and optimized for docking. The 3D crystal structures of KatG, MacB, and gidB were retrieved and prepared for molecular docking, molecular dynamics simulations, and ADMET profiling.

**Result:** Hesperidin showed the least binding energy (kcal/mol) against KatG (−9.3), MacB (−10.7), and gidB (−6.7); additionally, good pharmacokinetic profiles and structure–dynamics integrity with their respective protein complexes were observed.

**Conclusion:** Although these findings suggest hesperidin as a potential inhibitor against MacB, gidB, and KatG in *E. coli*, further validations through *in vitro* and *in vivo* experiments are needed. This research is expected to provide an alternative avenue for addressing existing antimicrobial resistances associated with *E. coli*’s MacB, gidB, and KatG.

## 1 Introduction

The growing problem of antibiotic resistance presents substantial risks to global public health and requires novel approaches to address the emergence of resistant bacterial strains (Lázár et al., 2013). *Escherichia coli* demonstrates the capacity of a bacterial pathogen and resists classical antimicrobial treatments by accumulating resistance mechanisms; its ongoing evolution of resistance mechanisms presents an opportunity for therapeutic intervention through identifying and targeting candidate genes related to resistance (Lázár et al., 2013). In this context, the utilization of the extensive genomic data on *E. coli*, along with prediction identification and development of inhibitors targeting resistance-induced candidate genes, presents a highly promising strategy for mitigating antibiotic resistance.

*E. coli* is a Gram-negative bacterium that is widely distributed in the environment and commonly found in the gastrointestinal tracts of both humans and warm-blooded animals (Carattoli et al., 2014). Although this is true for the majority of strains, some pathogenic variations have the potential to induce various diseases from relatively simple urinary tract infections to serious bloodstream infections (Carattoli, 2013). The urgent need for novel therapeutic compounds is highlighted by the growth of multidrug-resistant (MDR) bacteria, which are resistant to various classes of antibiotics. A comprehensive study of the genetic basis of antibiotic resistance in *E. coli* is therefore crucial for facilitating the advancement of antimicrobial medicines.

The use of whole-genome sequencing (WGS) has significantly transformed our capacity to unravel the genetic structures of bacterial pathogens, facilitating thorough examinations of their genomes with unparalleled precision (Didelot et al., 2012). By clarifying the genetic factors that contribute to antibiotic resistance, WGS enables identification of possible genes that may be targeted for suppression. Furthermore, comparative genomics enables investigation of evolutionary connections between the strains that are resistant and those that are susceptible, providing insights into the mechanisms driving the acquisition and spread of resistance (Didelot et al., 2012; Roemer et al., 2017).

The objective of this work is to utilize predictive modeling and computational methodologies to uncover potential genes that are involved in the resistance mechanisms within the *E. coli* genome. Our objective is to prioritize the candidate genes with the greatest potential for therapeutic interventions by integrating genomic, structural, and functional data. Moreover, *in silico* screening methodologies such as molecular docking, molecular dynamics (MD) simulations, and ADMET profiling are used in our objective to develop small molecule inhibitors that specifically target these potential genes. The ultimate aim of this work is to reinstate the ability to respond to antibiotic therapy.

This goal relies on the collaborative interactions among bioinformatics, computational biology, and medicinal chemistry, highlighting the interconnectedness of these fields in the pursuit of developing drugs to combat antibiotic resistance. By employing methodical and logical approaches, our objective is to accelerate the process of converting genetic knowledge into therapeutic interventions that are applicable in clinical settings. This aims to effectively tackle the increasing challenges presented by antibiotic-resistant strains of *E. coli*.

## 2 Materials and methods

### 2.1 Resistance gene and plasmid identifications

A total of 10 WGS datasets (FASTQ) of *E. coli* with identification numbers SRX19510069, SRX19510068, SRX19510067, SRX19510066, SRX19510065, SRX19510064, SRX19510063, SRX19510062, SRX19510061, and SRX19510060 were retrieved from the Sequence Read Archive (SRA; www.ncbi.nlm.nih.gov) maintained by the National Center for Biotechnology Information (NCBI). All data were filtered and clipped, and quality control procedures were implemented to remove low-quality reads and adapter sequences to improve the accuracy and reliability of downstream analyses. Using ResFinder (Bortolaia et al., 2020), the threshold for the percentage of identification of a resistance gene was set (≥90% identity over ≥60% of the length of the target gene). *E. coli* was selected as the chromosomal point mutation reference database, and antimicrobial configurations were used to select the desired antimicrobial resistance (AMR) genes.

**FIGURE 1:**
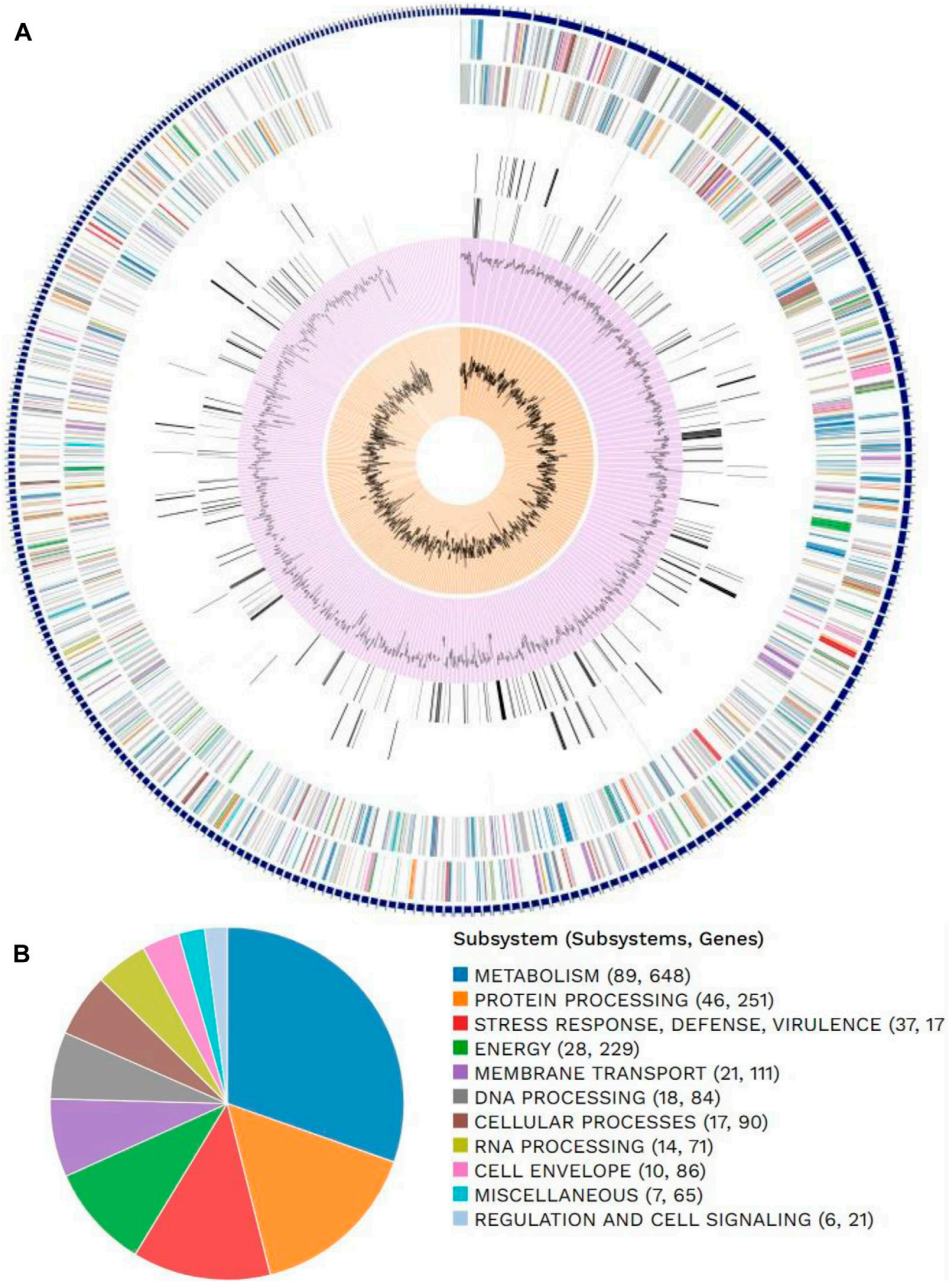
Distributions of **(A)** genome annotations and **(B)** subsystems in *Escherichia coli.*

The outputs were analyzed to define the AMR genotypes, i.e., patterns of resistance determinants observed for each antimicrobial substance in each dataset. The analyses and results visualization were carried out using R packages. PlasmidFinder 2.1 Database version (18-01-2023) was used to determine the plasmid replicon types of the assembled genomes of *E. coli*; the threshold for minimum percentage identity was 95%, and the minimum coverage of the contigs was set at 60%. pMLST 2.0 was used to determine the *in silico* plasmid MLST typing of the replicons and assembled genomes of *E. coli*, and the MLST configuration was set to IncF RST. Comprehensive genome analysis was carried out on the assembled data using the method described by Brettin et al. (2015), which was then annotated using the RAST tool kit (RASTtk) (Figure 1).

### 2.2 Plant compound sources and selection

A total of 42 compounds from different plants (Felipe et al., 2014; Thumann and Moissl-eichinger 2019; Dimitrijevi et al., 2021; Chen et al., 2022; Santhiravel et al., 2022) that have shown antibacterial properties against *E. coli* were chosen for the *in silico* evaluations. The control drugs for this project include isoniazid (PubChem ID: 3767) for KatG (PDB ID: 1U2J), chlorpromazine (PubChem ID: 2726) for gidB (PDB ID: 5LJ8), and sinefungin (PubChem ID: 65482) for MacB.

### 2.3 Selection and preparation of protein targets

The three-dimensional (3D) structures of the proteins for the KatG, MacB, and gidB of *E. coli* (Zhang et al., 2019) were modeled using the Swiss model server (https://swissmodel.expasy.org/). The proteins were made nascent by freeing them from heteroatoms, such as water molecules, ions, and ligands, followed by energy minimization using the protein preparation and minimization tools in Chimera^©^ software (version 1.13.1; https://www.cgl.ucsf.edu/chimera/) (Pettersen et al., 2004).

### 2.4 Preparation of compounds for molecular docking

The 3D conformers in the structure data files (SDFs) of the control drugs were obtained and downloaded from the PubChem chemical repository linked to the NCBI, which is one of the largest collections of freely accessible chemical information in the world. Further, the structures of the obtained compounds were converted to their best energetic and stable conformations through the Merck molecular force field (MMFF94) (Halgren, 1996) using the Open Babel tool and Python Prescription (version 0.8) (Umar et al., 2021).

### 2.5 Binding site predictions of KatG and MacB of *E. coli*

**FIGURE 2:**
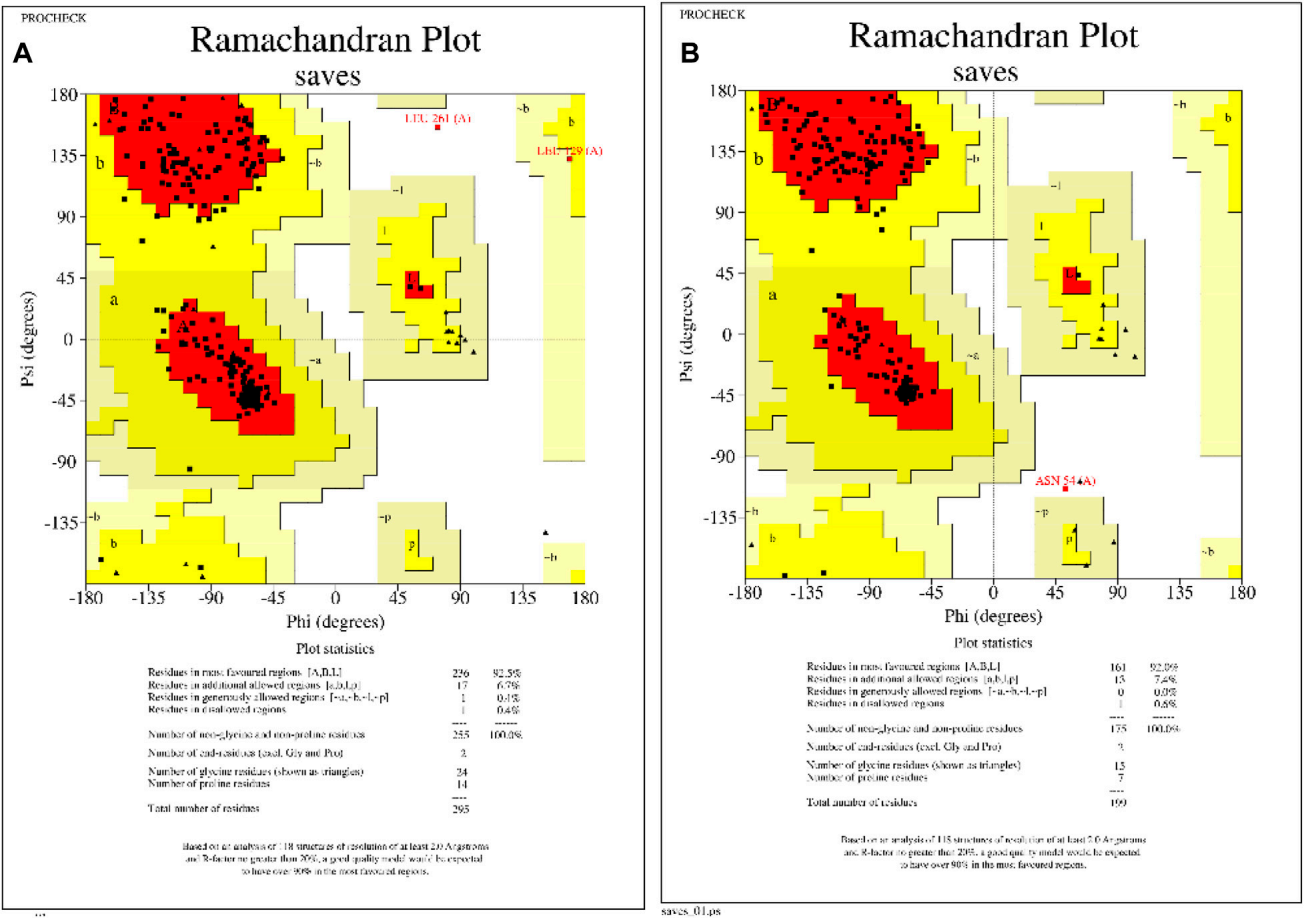
Homology modeled protein structural assessments of KatG and MacB genes of *E. coli.* **(A)** Ramachandran plot of KatG of *E. coli*, showing that 92.5% of the amino acid residues occupy the favored regions. **(B)** Ramachandran plot of MacB of *E. coli*, showing that 92% of the amino acid residues occupy the favored regions.

The prediction of KatG of E. *coli* using the protein-plus server (http://proteinsplus.zbh.uni-hamburg.de) (Volkamer et al., 2012) showed 11 possible binding pockets. The best binding pocket had a maximum drug score and a simple score, which were in agreement with the information supporting the server. After calculating the prediction, the P_0 pocket showed the best score for druggability and a simple score of 0.87 and 0.37, respectively. The same protein-plus server was used to predict the MacB of *E. coli,* for which 7 possible binding pockets were found. After running the prediction, the P_0 pocket was found to have the maximum drug score and a simple score of 0.82 and 0.63, respectively. These prediction outcomes are depicted in Figure 2 as well as Tables 4 and 5.

### 2.6 Molecular docking of compounds

Molecular docking was performed using AutoDock Vina and open-source Python Prescription 0.8 (Trott and Olson, 2010; Dallakyan and Olson, 2015) to acquire the possible orientations and binding energies (BEs) of the compounds at the binding sites of the proteins. A target region for KatG equivalent to the binding regions of the other monomers of the KatG gene was attuned with the aid of a grid box with dimensions 26.3979 × 11.2720 × 21.5337 Å, and the center was adjusted based on the sites of the monomer bindings in KatG comprising Leu48, Ser56, Arg58, Thr59, Thr62, Leu63, Ala66, Gly103, Ile104, Ala105, Arg107, Ala124, Val125, Val126, Ala128, Leu129, His134, Leu136, Gln137, Ala140, Asn164, Asn165, Val166, Asp220, Val221, Thr223, His259, Cys260, Leu261, Pro262, Val281, Gln284, Ala285, Arg288, Thr291, Ala292, Val295, Leu296, Leu299, and Leu300, which were obtained from the protein binding server Zentrum fur bioinformatik (https://proteins.plus/). A target region for MacB corresponding to the binding regions of the other monomers of the MacB gene was adjusted with the aid of a grid box with dimensions 22.1946 × 22.5720 × 26.3415 Å, and the center was adjusted based on the sites of the monomer bindings in MacB comprising Asp6, Tyr8, Pro9, Gly10, Lys11, Asp12, Phe13, Gly14, Asp15, Asp16, Ala44, Val45, Ser46, Gln47, Ala62, Asn63, Gly64, Val65, Tyr69, Tyr73, Met75, Glu132, Ser135, Met136, Phe137, Gly138, Ser139, Ser140, Lys141, Arg144, Trp146, Tyr149, Met152, Ser153, Trp161, Asn163, Ser164, Phe196, Trp198, and Met200, which were also obtained from the Zentrum fur bioinformatik server. A target region for gidB corresponding to the binding regions of the other monomers of the gidB gene was attuned with the aid of a grid box with dimensions 26.3979 × 11.2720 × 26.3415 Å, and the center was adjusted based on the sites of the monomer bindings in gidB including Asn35, Asp71, Gly73, Gly77, Leu78, Asp96, Ser97, Leu98, Arg101, and Arg123 (Zhang et al., 2019). After the docking runs, the three compounds with the best docking scores (binding energies) below those of the control drugs were subjected to molecular visualization to analyze their molecular interaction fingerprints using PyMOL^©^ Molecular Graphics (version 2.4, 2016, Schrödinger LLC) (Seeliger and Groot, 2010) and Maestro (version 12.5, 2016, Schrödinger LLC). The molecular interaction fingerprints analyzed for each protein–ligand complex included hydrogen bonds, hydrophobic interactions, and electrostatic linkages between the amino acids of the protein and ligand atoms.

### 2.7 ADMET prediction *in silico*

The absorption, distribution, metabolism, excretion, and toxicity (ADMET) are important at the early stages of drug discovery and design pipeline to analyze the pharmacokinetics of the proposed compounds that could serve as drugs. The ADMETSar server was used to forecast the ADMET properties of the compounds with the best hits after molecular docking analysis (Cheng et al., 2012; Yang et al., 2018). The server was fed with the SMILE strings of the compounds from PubChem (https://pubchem.ncbi.nlm.nih.gov/compound/) through the search bar to predict the ADMET properties.

### 2.8 MD simulations

The three targets complexed with hesperidin served as the initial structures for the MD simulations in the Desmond package (Schrödinger Release 2019-3) based on the method described by Tutumlu et al. (2020). The simulations were performed for 200 ns durations, and various parameters were carefully examined to assess the structural stabilities of the tested compounds. To ascertain seamless amalgamation with the Schrodinger interface, the starting structures underwent meticulous preparation exploiting the Protein Preparation Wizard. Multiple crucial tasks were carried out to achieve the research objectives, including adding hydrogen atoms, assigning bond orders, and addressing any absent amino acid side chains and loops by optimizing the hydrogen bond assignments. Additionally, water orientation sampling at pH 7.0 was conducted. Through the System Builder option, the simulation periodic box was created, and solvation was accomplished by engaging the all-atom force field optimized potentials for liquid simulations (OPLSs) in combination with the single point charge (SPC) water model. An exhaustive system minimization was conducted, involving 1,000 iterations of the steepest descent technique and equilibration under the constant number of atoms, pressure, and temperature (NPT) ensemble conditions. The equilibration was implemented for 200 ns at a temperature of 300 K and pressure of 1.01325 bar. To regulate the temperature during the simulations, the Nosé–Hoover thermostat with a relaxation time of 1 ps was used, while the isotropic Martyna–Tobias–Klein barostat with a relaxation time of 2 ps was used to maintain constant pressure. Short-range interactions were considered with a cutoff of 9 Å, while the smooth particle mesh Ewald (PME) method was used in combination with the reversible reference system propagator algorithm (RESPA) integrator to calculate the long-range Coulombic interactions. To capture the dynamics of the system successfully, conformational snapshots were exported at regular intervals of 5 ps during the course of the simulations. At the end of the simulations, systemic stability was appraised using root mean-squared deviations (RMSDs), root mean-squared fluctuations (RMSFs), and assessments of the protein–ligand contacts. These analyses provided valuable insights into the behaviors and interactions of the protein–ligand complexes studied.

### 2.9 Principal component analysis (PCA)

PCA was conducted through the ProDy library (version 1.5.1) within Python (www.python.org). This mainly involves aligning the structures based on the Cα atoms, which was accomplished using ProDy’s iterative superposition approach. The covariance matrix was calculated from the ensemble, and its diagonalization yielded the principal components, which were saved in ProDy’s NMD file format (Bakan et al., 2011). Notably, the analysis solely considered the Cα atoms, while the gaps were assigned weights of 0.0.

### 2.10 Dynamic cross-correlation (DCC) analysis

The DCC maps were constructed for the Cα atoms from the concatenated trajectories with respect to the reference structures using the “Bio3D” option in the R- and ProDy-based analysis tool (Grant et al., 2021).

### 2.11 Free energy landscape (FEL) analysis

For each of the selected models, we produced free energy landscapes (FELs) with respect to the radius of gyration (Rg) and RMSD trajectories using the Geo-Measures plugin in the PyMOL package (Kagami et al., 2020).

## 3 Results and discussion

### 3.1 Genome assembly details

The assembly details outlined in Table 1 offer a comprehensive view of the genomic characteristics under study. With a total of 478 contigs, the genome appears to be somewhat fragmented, necessitating further investigations into potential scaffoldings or misassemblies. The GC content of 50.68% falls within the expected range for many organisms and serves as a baseline indicator of the nucleotide composition. Notably, the absence of plasmids in the assembly suggests a lack of extrachromosomal genetic elements in the sampled organism.

**TABLE 1:** Details of the genome assembly.

S/No.	Genome properties	Value
1	Contigs	478
2	GC Content (%)	50.68
3	Plasmids	0

### 3.2 Annotated genome features

Table 2 presents the annotated genome features of the whole-genome analysis of *E. coli*, which contains 3,456 coding sequences (CDSs) demonstrating the vast genetic information stored within its DNA as well as 20 transfer RNA (tRNA) that are required for protein syntheses and cellular processes. The abundance of CDSs within *E. coli*’s genomic makeup is particularly important in drug discovery because it represents a rich source of potential drug targets. However, the current work is primarily in the interest of those implicated in antibiotic resistance.

**TABLE 2:** Features from the genome annotation.

S/No.	Genome properties	Value
1	Coding sequence (CDS)	3,456
2	tRNA	20
3	Repeat regions	4
4	Partial CDS	0
5	rRNA	0
6	Miscellaneous RNA	0

### 3.3 AMR genes

Table 3 presents the various AMR genes discovered from the whole-genome analysis of *E. coli*; these genes highlight the diverse strategies employed by *E. coli* to resist the effects of antibiotics. Notably, the presence of genes encoding the antibiotic activation enzyme KatG suggests a sophisticated ability to modify or neutralize antibiotics. The presence of antibiotic resistance gene clusters, such as MarA, MarB, and MarR, further underscores the complexity of the genetic basis for AMR in this pathogen; these genes that are associated with antibiotic targets in susceptible species and those conferring protection to these targets (e.g., BcrC) suggest a nuanced approach to evading the effects of antibiotics at the molecular level. Moreover, the presence of genes related to efflux pump systems (e.g., AcrAB-TolC, AcrZ, EmrAB-TolC, MacA, MacB, MdfA/Cmr, MdtEF-TolC, MdtL, and TolC/OpmH) indicates an active role in expelling antibiotics from the bacterial cell, contributing to a broader spectrum of resistance mechanisms. The presence of gidB, which contributes to resistance via absence, adds another layer of intricacy to the genetic landscape of antibiotic resistance in *E. coli*. Additionally, the involvement of the protein altering cell wall charge (PgsA) and regulators modulating the expressions of antibiotic resistance genes (e.g., AcrAB-TolC, EmrAB-TolC, GadE, H-NS, and OxyR) suggests a coordinated and adaptive response to antibiotic exposure. The diverse array of AMR genes identified in *E. coli* shows the urgent need for a drug with a multifaceted approach or mechanism of action in addressing antibiotic resistance in this pathogen.

**TABLE 3:** Potential drug targets in *Escherichia coli* that confer antibiotic resistance.

S/No	Antimicrobial resistance mechanism	Genes/proteins
1	Antibiotic activation enzyme	KatG
2	Antibiotic resistance gene cluster, cassette, or operon	MarA, MarB, MarR
3	Antibiotic target in susceptible species	Alr, Ddl, dxr, EF-G, EF-Tu, folA, Dfr, folP, gyrA, gyrB, inhA, fabI, Iso-tRNA, MurA, rho, rpoB, rpoC, S10p, S12p
4	Antibiotic target protection protein	BcrC
5	Efflux pump conferring antibiotic resistance	AcrAB-TolC, AcrZ, EmrAB-TolC, MacA, MacB, MdfA/Cmr, MdtEF-TolC, MdtL, TolC/OpmH
6	Gene conferring resistance via absence	gidB
7	Protein altering cell wall charge conferring antibiotic resistance	PgsA
8	Regulator modulating expression of antibiotic resistance genes	AcrAB-TolC, EmrAB-TolC, GadE, H-NS, OxyR

### 3.4 Homology modeling of *E. coli*’s KatG and MacB genes

The 3D protein structures of *E. coli*’s KatG and MacB have been crystallized and deposited in the popular RCSB protein database but have numerous missing residues and loops. Therefore, the homology modeling approach was employed to build suitable protein structures for this study. After building the structures, the models were assessed and showed that the KatG and MacB modeling for *E. coli* had 92.5% identity and 92.0% of the amino acid residues occupying the favored region, respectively (Figure 2). These indicate that the modeled structures are suitable for molecular docking and MD simulations.

### 3.5 Binding site predictions of KatG and MacB

**FIGURE 3:**
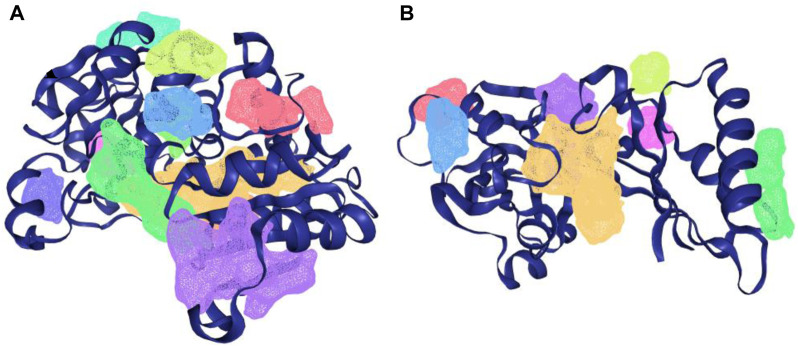
Density map presentation of the predicted binding sites of KatG and MacB of *E. coli* using the DoGsiteScorer module of protein-plus server (http://proteinsplus.zbh.uni-hamburg.de). **(A)** Density map representation of the predicted binding sites of KatG of *E. coli*, with the best binding site shown in purple. **(B)** Density map representation of the predicted binding sites of MacB of *E. coli*, with the best binding site shown in yellow.

The protein-plus server was used to identify 11 and 7 possible binding pockets for KatG and MacB, respectively (Tables 4, 5). The best binding pocket is the one with the maximum score for druggability and simple score; this is in accordance with the information supporting the server (Volkamer et al., 2012). After implementing the predictions, the P_0 pockets for both proteins were found to be the best choices as they had the maximum drug scores and simple scores each. The density map representations of the predicted pockets of both proteins are presented in Figure 3.

**TABLE 4:** Binding sites predicted from KatG of E. *coli* using the protein-plus server (http://proteinsplus.zbh.uni-hamburg.de).

Name	Volume Å³	Surface Å^2^	Drug score	Simple score
P_0	640.77	617.29	0.87	0.37
P_1	628.67	967.66	0.72	0.47
P_10	106.88	62.72	0.38	0
P_2	352.13	718.09	0.8	0.25
P_3	229.38	423.13	0.62	0
P_4	176.26	248.18	0.39	0.02
P_5	145.86	318.6	0.36	0
P_6	141.31	297.76	0.27	0.01
P_7	134.53	376.65	0.37	0
P_8	128.64	300.67	0.27	0
P_9	114.88	255.86	0.19	0

**TABLE 5:** Binding sites predicted from MacB of *E. coli* using the protein-plus server (http://proteinsplus.zbh.uni-hamburg.de).

Name	Volume Å³	Surface Å^2^	Drug score	Simple score
P_0	941.5	910.51	0.82	0.63
P_1	378.24	697.83	0.79	0.23
P_2	141.25	357.84	0.4	0.06
P_3	129.34	257.75	0.24	0
P_4	125.57	309.59	0.24	0
P_5	107.01	287.29	0.13	0
P_6	106.82	301.5	0.28	0

### 3.6 Binding abilities

Three out of the 42 compounds docked against the protein targets displayed better binding potentials based on their BEs (between −6.5 and −6.7 kcal/mol for gidB than −5.2 kcal/mol for sinefungin; between −8.5 and −9.4 kcal/mol for KatG than −5.4 kcal/mol for isoniazid, and −10.7 kcal/mol for MacB than −7.9 kcal/mol for chlorpromazine). The details for the 42 compounds and three targets are provided in Table 6. Hesperidin (−9.3 kcal/mol), rutin (−9.1 kcal/mol), and naringin (−8.8 kcal/mol) returned the least BEs against KatG. Similarly, biacalein (−6.7 kcal/mol), chrysin (−6.5 kcal/mol), and hesperidin (−6.7 kcal/mol) had the least BEs against gidB. Finally, epicatechin gallate (−10.7 kcal/mol), hesperidin (−10.7 kcal/mol), and epigallocatechin gallate (−10.7 kcal/mol) had the least BEs against MacB. Interestingly, hesperidin was able to bind all three targets in *E. coli* that are implicated in antibiotic resistance through different pathways (Table 6).

**TABLE 6:** Binding energies (kcal/mol) of compounds docked against gidB, KatG, and MacB of *E. coli* using AutoDock Vina.

S/N	Compound name	PubChem ID	Binding energy (kcal/mol)		
			gidB	KatG	MacB
1	Hesperidin	10621	−6.7	−9.3	−10.7
2	Syringic acid	10742	−4.4	−5.5	−6.5
3	(−)-Epicatechin gallate	107905	−5.6	−8.5	−10.7
4	Daidzin	107971	−5.5	−8.1	−10
5	Astilbin	119258	−5.2	−8.4	−7.5
6	2-Chloro-3-(4-hydroxyphenyl)-1-propene	128853	−5.7	−7.6	−9.1
7	Cyanidin	128861	−5.5	−7.4	−8.9
8	4-Hydroxybenzoic acid	135	−4.7	−5	−6.3
9	Malvidin	159287	−5.3	−6.7	−7.9
10	Chlorogenic acid	1794427	−5.7	−7.5	−9.6
11	Gallic acid	370	−4.5	−5.6	−6.5
12	Control drug (isoniazid)	3767	—	−5.1	—
13	Naringenin	439246	−6.2	−7	−9.1
14	Taxifolin	439533	−5.8	−7.1	−9.3
15	Pelargonidin	440832	−5.4	−6.9	−9.1
16	Peonidin	441773	−5.3	−6.7	−9.1
17	Petunidin	441774	−5.2	−7.2	−9.1
18	Naringin	442428	−6.1	−8.8	−9.8
19	Resveratrol	445154	−5.3	−6.6	−8.6
20	Ferulic acid	445858	−5	−5.6	−7.1
21	Quercetin	5280343	−5.5	−7.1	−9.2
22	Apigenin	5280443	−6.4	−6.9	−9.1
23	Luteolin	5280445	−5.6	−7.2	−9.2
24	Rutin	5280805	−5.3	−9.1	−7.9
25	Kaempferol	5280863	−5.6	−6.7	−9.3
26	Genistein	5280961	−5.4	−7.0	−9.0
27	Baicalein	5281605	−6.7	−7.0	−9.8
28	Chrysin	5281607	−6.5	−6.9	−9.8
29	Isorhamnetin	5281654	−5.4	−6.9	−8.7
30	Myricetin	5281672	−5.8	−7.4	−9.4
31	Daidzein	5281708	−5.4	−6.8	−8.9
32	Glycitein	5317750	−5.4	−6.7	−8.4
33	p-Coumaric acid	637542	−4.9	−5.5	−6.9
34	Sinapic acid	637775	−4.6	−5.9	−7.3
35	Engeletin	6453452	−5	−8	−8.2
36	(−)-Epigallocatechin gallate	65064	−5.7	−8.3	−10.7
37	Piceatannol	667639	−5.1	−6.9	−8.6
38	Caffeic acid	689043	−5.2	−5.9	−7
39	(−)-Epicatechin	72276	−5.6	−7.7	−8.8
40	Epigallocatechin	72277	−5.8	−7.7	−8.5
41	Hesperetin	72281	−5.8	−7.2	−8.6
42	3,4-Dihydroxybenzoic acid	72	−5.1	−5.2	−6.5
43	4-Chloro-3-nitrobenzotrifluoride	8468	−4.9	−5.1	−6.6
44	Control drug (sinefungin)	65482	−5.2	—	—
45	Control drug (chlorpromazine)	2726	—	—	−6.5

### 3.7 Binding positions and molecular interaction imprints

**FIGURE 4:**
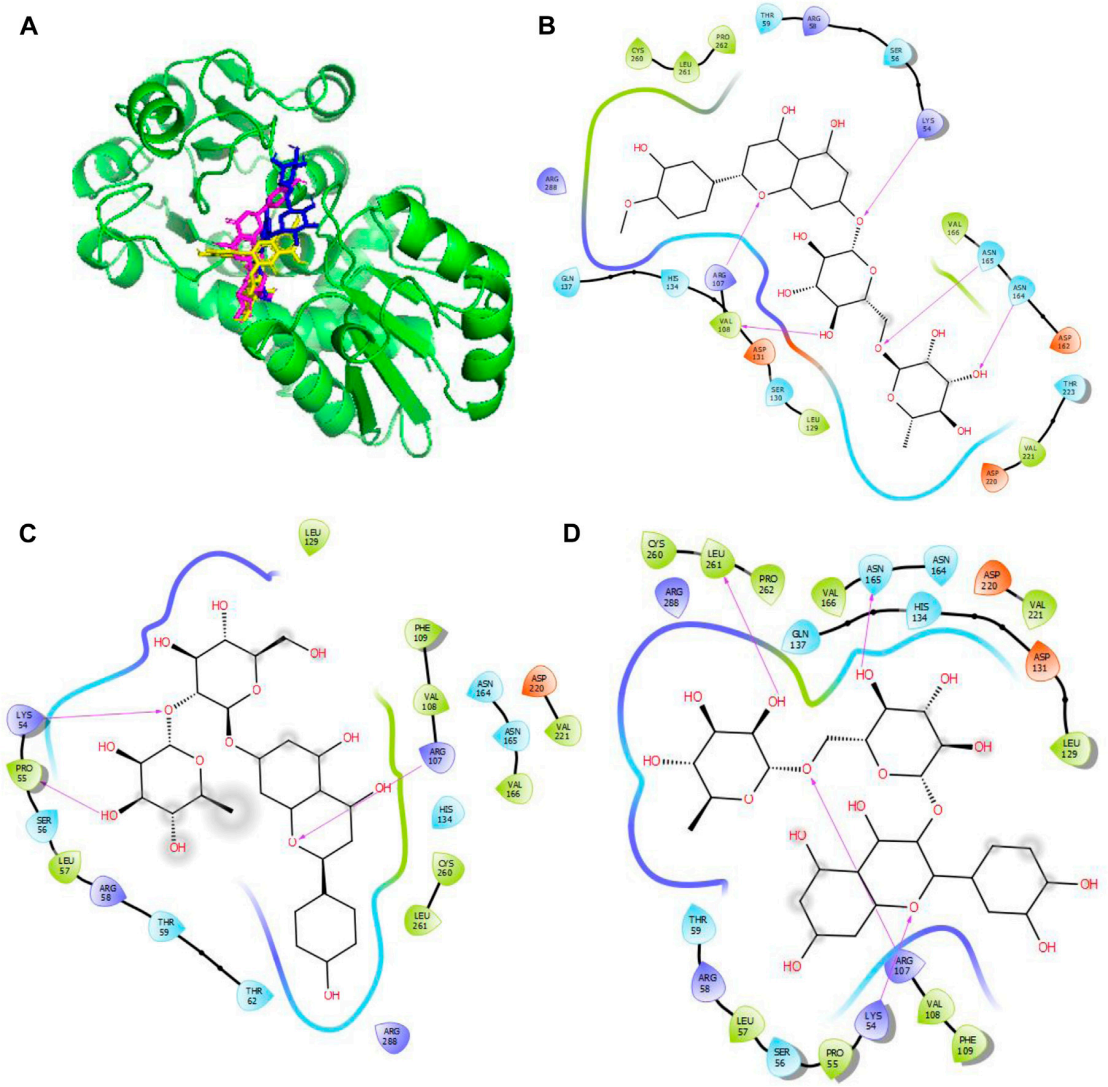
Molecular docking of the bioactive compounds in KatG of *E. coli.* **(A)** 3D binding positions of KatG (green), hesperidin (blue), naringin (purple), and rutin (yellow) in the binding pockets of KatG of *E. coli*. 2D molecular interaction analyses of **(B)** hesperidin, **(C)** naringin, and **(D)** rutin with the amino acid residues in the binding pockets of KatG of *E. coli*.

**FIGURE 5:**
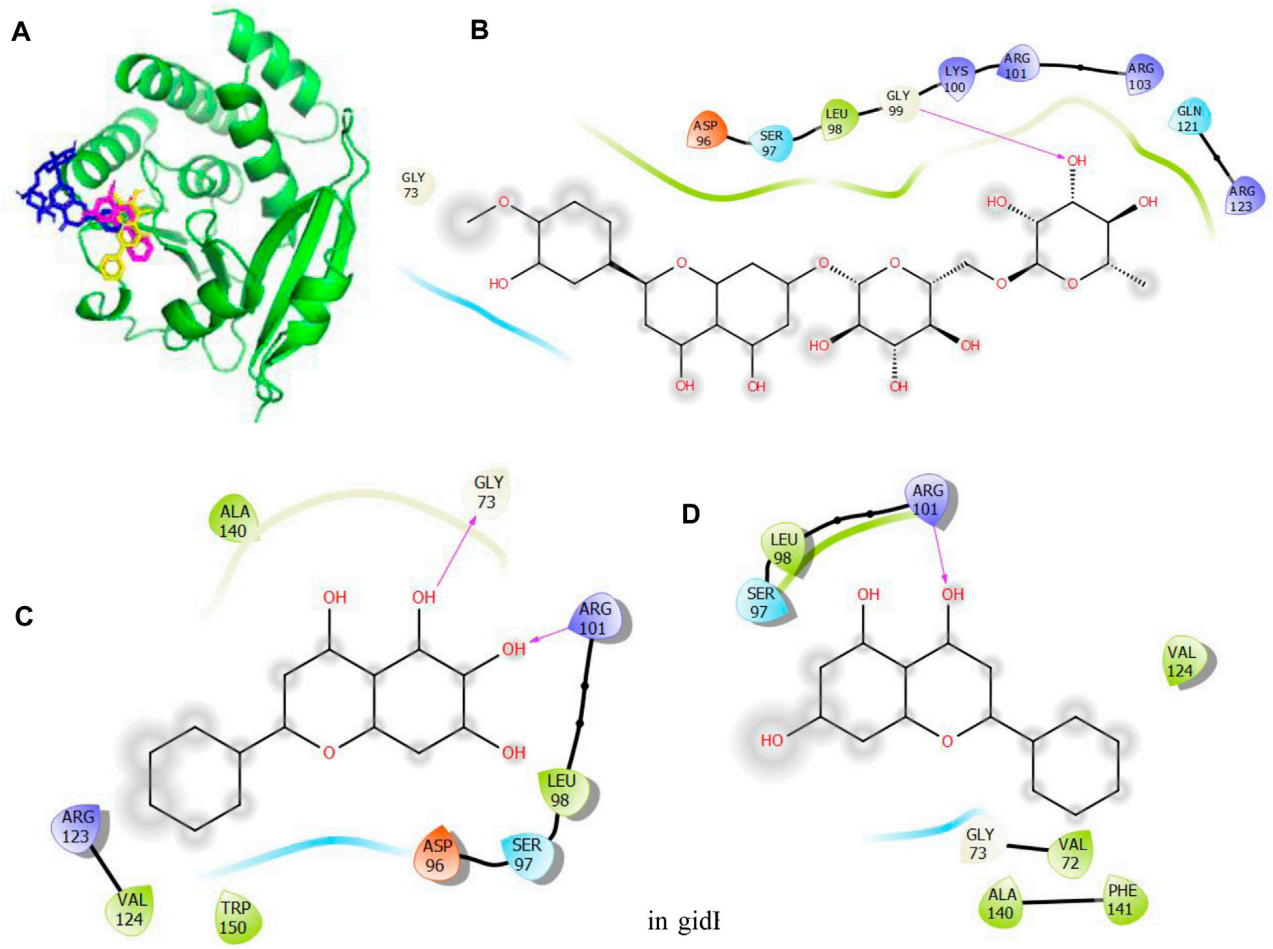
Molecular docking of the bioactive compounds in gidB of *E. coli.* **(A)** 3D binding positions of gidB (green), hesperidin (blue), chrysin (purple), and baicalein (yellow) in the binding pockets of gidB of *E. coli*. 2D molecular interaction analyses of **(B)** hesperidin, **(C)** baicalein, and **(D)** chrysin with the amino acid residues in the binding pockets of gidB of *E. coli*.

**FIGURE 6:**
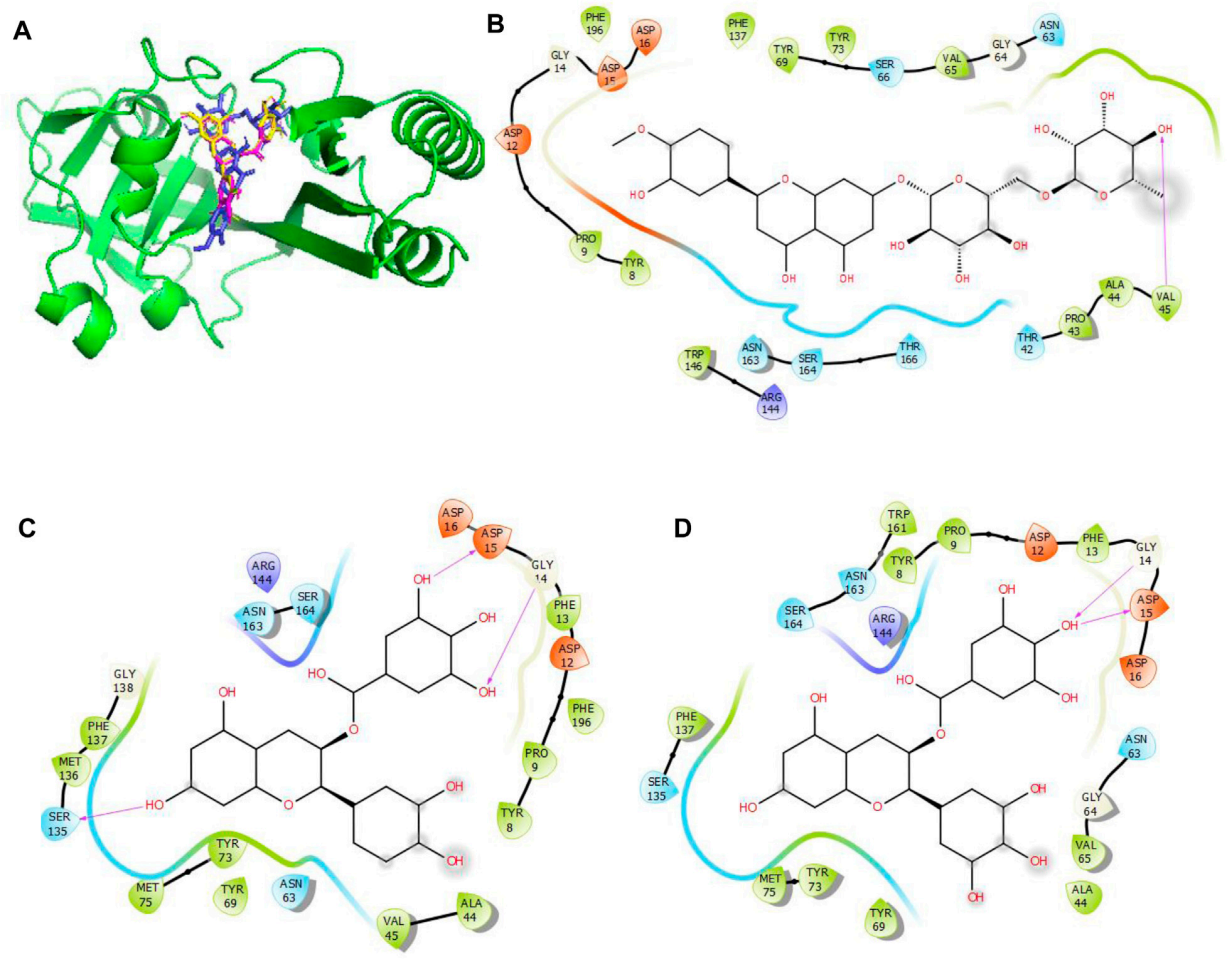
Molecular docking of the bioactive compounds in MacB of *E. coli.* **(A)** 3D binding positions of MacB (green), hesperidin (blue), epigallocatechin gallate (purple), and epicatechin gallate (yellow) in the binding pockets of MacB of *E. coli*. 2D molecular interaction analyses of **(B)** hesperidin, **(C)** epicatechin gallate, and **(D)** epigallocatechin gallate with the amino acid residues in the binding pockets of MacB of *E. coli*.

Given the BEs observed in Table 6, there is a need to further probe the mechanisms involved in the binding of these compounds with the target proteins. These indicate the relevant relationships between the protein–compound complexes of these compounds. From a recent work, hydrogen bonds and hydrophobic interactions were majorly observed to occur between the atoms of the compounds and side chains of the amino acid residues occupying the binding sites in KatG, MacB, and gidB of E. *coli*. These details are provided in Figures 4–6.

In Figure 4, the binding positions show that the three compounds occupy the same areas at the binding sites of KatG. It was observed that hydrogen bonds were established between the three compounds as well as Lys54 and Arg107. Aside from these two amino acid residues, Val108, Asn165, and Asn164 were found to link with hesperidin via hydrogen bonding, while Leu261 and Asn165 were involved with rutin via hydrogen bonding. Finally, Pro55 was the only extra amino acid to interact with naringin via hydrogen bonds. It was also observed that all three compounds interacted with Cys260, Thr59, Arg58, Ser56, Arg288, His134, Leu129, Asp220, Val221, and Val166 via hydrophobic interactions. Interestingly, four out of the six amino acids (Arg107, Asn164, Leu261, and Ans165) that interacted with the chosen compounds via hydrogen bonds were earlier predicted to be members of the binding site residues. Moreover, nine out of ten amino acids that interacted with the compounds were among the predicted amino acids of the binding sites.

In Figure 5, the binding positions show that the three compounds occupy the same areas at the binding sites of gidB. It was observed that hydrogen bonds were established between the atoms of biacalein as well as Gly73 and Arg101, between chrysin and Arg101, and between hesperidin and gly99. In addition, all three compounds interacted with Ala140, Leu98, Ser97, Asp96, Trp150, Val124, Arg123, Val72, Gly73, Phe141, Gln121, and Arg103 via hydrophobic interactions. Interestingly, Gly73 and Arg101 that interacted with the chosen compounds via hydrogen bonds were earlier predicted to be members of the binding site residues, while Asp96, Gly73, Arg123, Leu98, and Ser97 that interacted with the compounds via hydrophobic interaction were among the predicted amino acids of the binding sites.

In Figure 6, the binding positions show that the three compounds occupy the same areas at the binding sites of MacB. It was observed that hydrogen bonds were established between the atoms of epicatechin gallate as well as Ser135, Gly14, and Asp15; between epigallocatechin gallate as well as Gly14 and Asp15; and finally between hesperidin and Val45. In addition, all three compounds interacted with Asp12, Arg144, Asn163, Ser164, Tyr8, Pro9, Phe137, Tyr73, Tyr69, Asn63, and Ala44 via hydrophobic interactions. Interestingly, Asp15, Gly14, Ser135, and Val45 that interacted with the chosen compounds via hydrogen bonds were earlier predicted to be among the binding site residues, while Asp12, Ser164, Tyr8, Pro9, Phe137, Ala44, Tyr69, Asn63, and Ala44 that interacted with the compounds via hydrophobic interactions were among the predicted amino acids of the binding sites.

### 3.8 ADMET predictions *in silico*

The ADMET predictions were carried out to ascertain how the three chosen compounds for each of the proteins were absorbed, distributed, metabolized, and eliminated as well as how toxic they can be. The outcomes of the evaluations are presented in Table 7. Hesperidin, epicatechin gallate, chrysin, and naringin showed good ADMET profiles and do not tend to cause mutations against *Salmonella typhenurium* (AMES). Epigallocatechin gallate and rutin tend to cause mutations against *S. typhenurium* (AMES). Rutin was observed to be a non-inhibitor of the human ether-a-go-go (herG) gene, while the other compounds were inhibitors to the human herG gene. Correspondingly, all these compounds did not display the ability to cross the blood–brain barrier (BBB) and may not be absorbed through caco-2. They tend to have good intestinal absorptions except for hesperidin and naringin. None of the compounds were predicted to serve as substrates to P-glycoprotein, and all the compounds were found to be substrates to cytochrome P450 isoform 3A4 except biacalein and chrysin.

**TABLE 7:** ADMET properties of the three best compounds selected from compounds docked against the three proteins implicated in antibiotic resistance in *E. coli* using ADMETSar server.

ADMET profiles	Naringin	Isoniazide^*^	Epicatechin gallate	Epigallocatechin gallate	Chlorpromazine^**^
Ames mutagenesis	−	+	−	+	−
Acute oral toxicity (class)	III	III	IV	IV	II
Blood–brain barrier	−	+	−	−	+
Caco-2	−	+	−	−-	+
Carcinogenicity	−	−	−	−	−
CYP1A2 inhibition	−	+	−	−	+
CYP2C19 inhibition	−	−	−	−	+
CYP2C8 inhibition	+	−	+	+	−
CYP2C9 inhibition	−	−	−	−	−
CYP2C9 substrate	−	−	−	−	−
CYP2D6 inhibition	−	−	−	−	+
CYP2D6 substrate	−	−	−	−	+
CYP3A4 inhibition	−	−	−	−	−
CYP3A4 substrate	+	−	+	+	+
CYP inhibitory promiscuity	−	−	−	−	+
Hepatotoxicity	−	+	+	+	+
Human ether-a-go-go-related gene inhibition	+	−	+	+	+
Human intestinal absorption	−	+	+	+	+
Human oral bioavailability	−	+	−	−	−
Acute oral toxicity	1.980874	1.764945	2.094668	2.432732	2.261878
P-glycoprotein inhibitor	−	−	−	−	+
P-glycoprotein substrate	−	−	−	−	+
Plasma protein binding	0.672338	0.176502	0.96188	1.039135	0.930981
Subcellular localization	Mitochondria	Mitochondria	Mitochondria	Mitochondria	Lysosomes
UGT catalyzed	+	−	−	−	−
Water solubility	−2.53371	−0.05213	−3.3141	−3.3141	−4.94739

ADMET profiles	Baicalein	Chrysin	Rutin	Sinefungin^***^	Hesperidin
Ames mutagenesis	−	−	+	−	−
Acute oral toxicity (class)	II	III	III	III	III
Blood–brain barrier	−	−	−	−	−
Caco-2	−	+	−	−	−
Carcinogenicity	−	−	−	−	−
CYP1A2 inhibition	+	+	−	−	−
CYP2C19 inhibition	−	+	−	−	−
CYP2C8 inhibition	+	+	−	−	+
CYP2C9 inhibition	−	+	−	−	−
CYP2C9 substrate	−	−	−	−	−
CYP2D6 inhibition	−	−	−	−	−
CYP2D6 substrate	−	−	−	−	−
CYP3A4 inhibition	+	+	+	−	−
CYP3A4 substrate	−	−	−	−	+
CYP inhibitory promiscuity	+	+	−	−	−
Hepatotoxicity	−	−	+	−	−
Human ether-a-go-go-related gene inhibition	−	−	−	−	+
Human intestinal absorption	+	+	+	+	−
Human oral bioavailability	+	−	−	−	−
Acute oral toxicity	2.210606	1.92548	2.285344	2.039988	1.953582
P-glycoprotein inhibitor	−	−	−	−	−
P-glycoprotein substrate	−	−	−	−	−
Plasma protein binding	1.024719	0.947877	0.901161	0.064035	0.852145
Subcellular localization	Mitochondria	Mitochondria	Mitochondria	Mitochondria	Mitochondria
UGT catalyzed	+	+	+	−	+
Water solubility	−2.99937	−2.77654	−2.7724	−2.22868	−2.64853

*, **, and *** control drugs.

The toxicities of the chosen compounds ranged between classes II to IV. In addition, they were predicted to be localized in the mitochondria. Biacalein, chrysin, hesperidin, and naringin were predicted to undergo phase-two drug metabolisms through glucorunidation. However, studying their structures keenly for the purpose of generating chemosimilars with improved safety profiles may be of great relevance and should be considered as a subject of future investigation.

### 3.9 Dynamics stabilities of the protein–ligand complexes

Hesperidin (hesperetin-7-O-rutinoside), which shows great affinity across the three protein targets, is a flavone made up of the rhamnose, glucose, and hesperetin aglycone moieties. It is abundantly available in citrus plants and is majorly produced in the citrus industry (Rudrapal et al., 2023). According to recent preclinical and clinical studies on its biological usage as an active element, it possesses antioxidant, anti-inflammatory, lipid-lowering, and insulin sensitivity properties as well as potency in neurological disorders, psychiatric disorders, and cardiovascular diseases because of its effects on the blood pressure (Atoki et al., 2023; Hosawi, 2023; Ileriturk et al., 2023).

**FIGURE 7:**
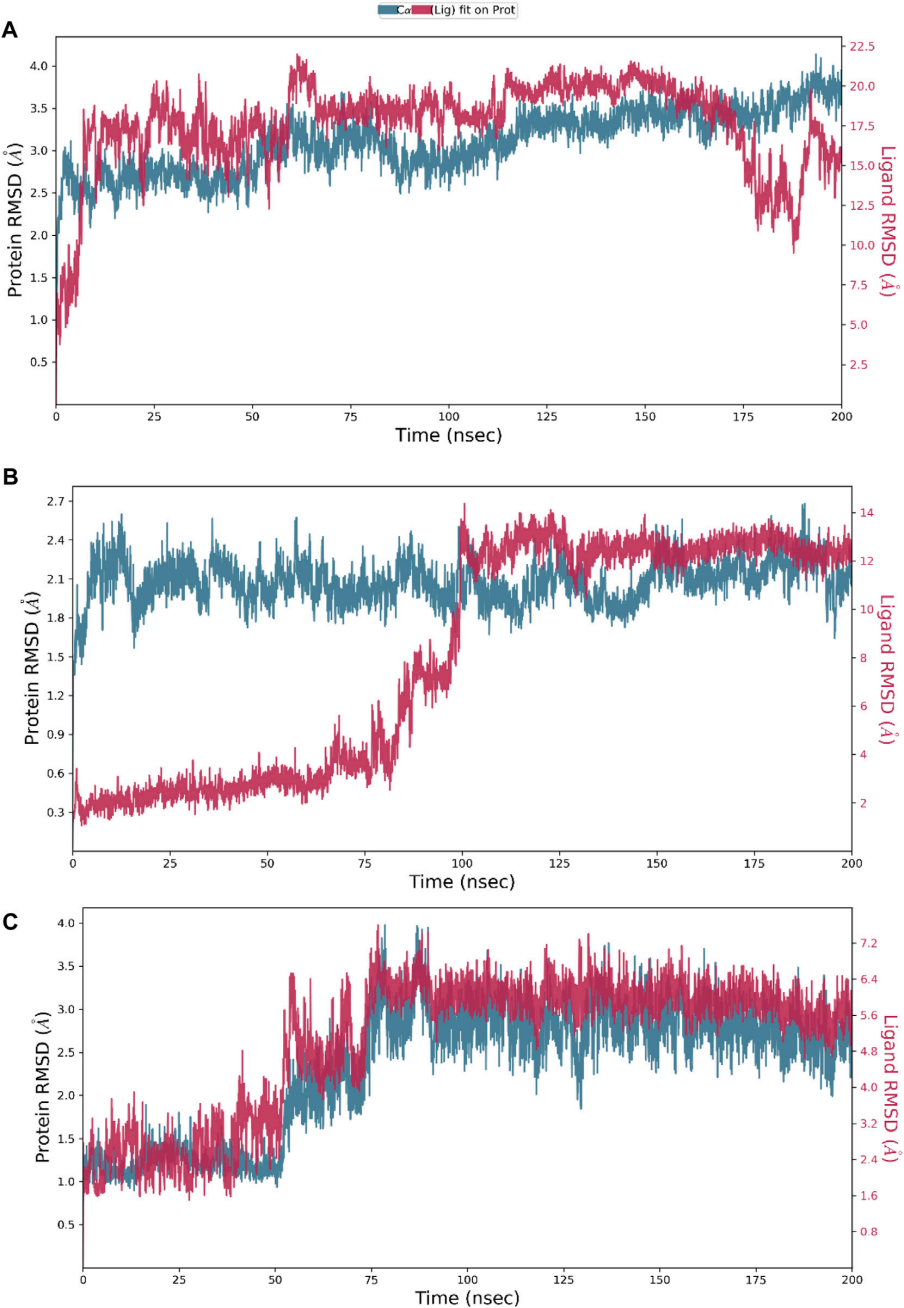
Dynamic stability metrics of hesperidin-bound protein complexes over 200 ns of molecular dynamics (MD) simulations. Time-dependent RMSD profiles of the backbone atoms of **(A)** hesperidin–gidB, **(B)** hesperidin–KatG, and **(C)** hesperidin–MacB complexes.

**FIGURE 8:**
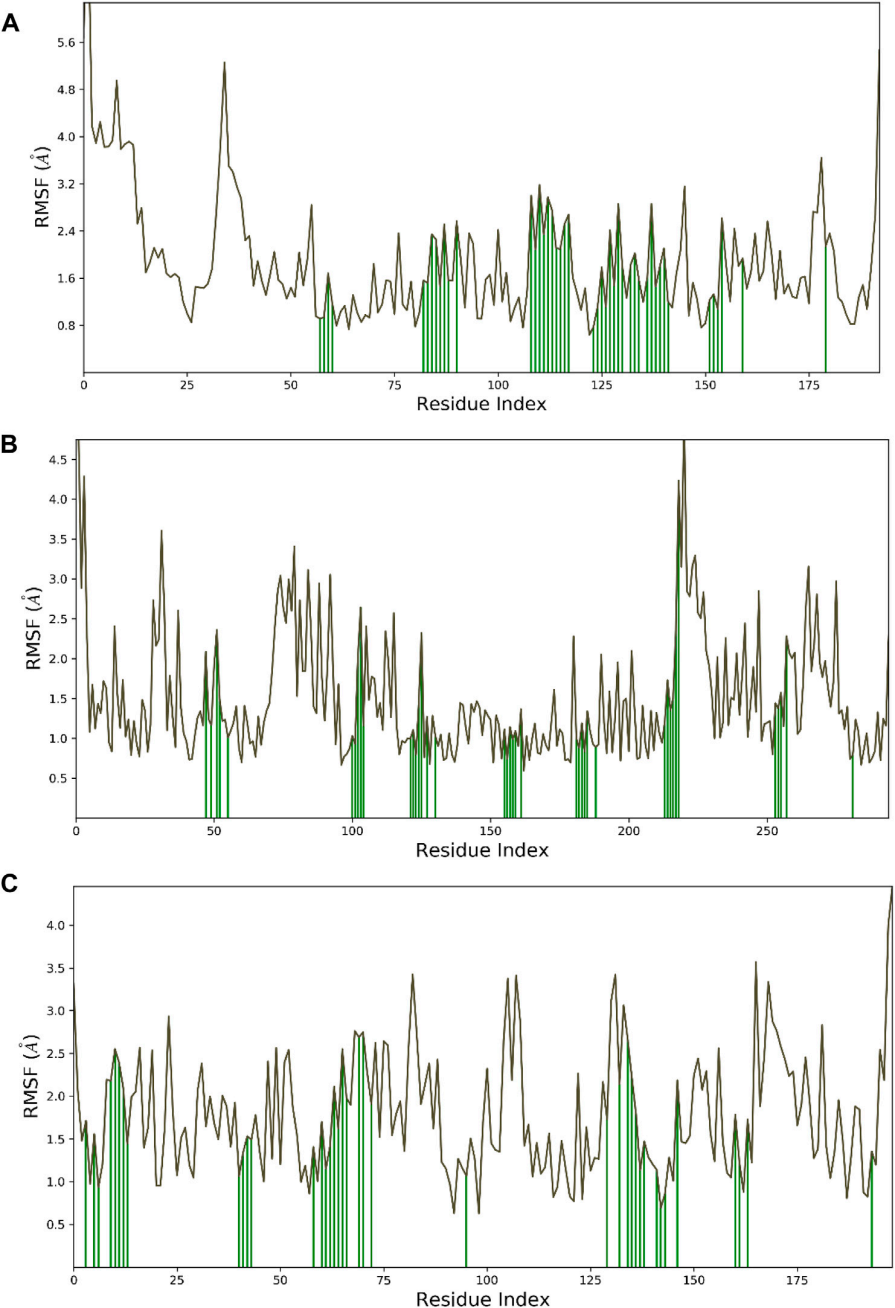
Dynamic stability metrics of hesperidin-bound protein complexes over 200 ns of MD simulations. Cα-RMSF profiles of the **(A)** hesperidin–gidB, **(B)** hesperidin–KatG, and **(C)** hesperidin–MacB complexes. The green lines indicate the points of contact of hesperidin with the amino acids during the MD runs.

Hesperidin was found to have good binding abilities against the three selected protein targets in this work. Therefore, a unique study engaging MD simulation was undertaken for the three complex systems to delve into the structural and dynamic behaviors of gidB, KatG, and MacB in the presence of hesperidin. The study focused on investigating the intrinsic dynamic stability of each complex by analyzing their backbone RMSD (Figure 7) and RMSF (Figure 8) in 200 ns simulations.

The RMSD serves as a measure of variance between a protein backbone from its starting to final conformations and offers beneficial insights into the stability of the protein–ligand complex (Cavasotto et al., 2019; Solo-Aben et al., 2021; Danazumi and Umar, 2023). A plot of divergences observed during the simulations was used to measure the relative stabilities, whereby more negligible deviations indicate more stable protein structures (Hollingsworth and Dror, 2018; Tran et al., 2022). The RMSD values for the backbones were calculated via 200 ns simulations to establish the stabilities of the three systems. At the early stage of the simulation, the hesperidin–gidB complex (Figure 7A) exhibited an average RMSD (indicated in red) of 3.5 Å from 0 ns to 60 ns. From 100 ns to 175 ns, equilibrium was reached, as shown in the trajectory plot, followed by a gradual decline, reaching an average RMSD peak of 2.0 Å at 180 ns. Subsequently, the curve rises to 3.5 Å at 190 ns and is stabilized for the next 10 ns before declining to 2.4 Å and remaining stable until the end of the simulation period. A keen inspection of the gidB complex system revealed a moderately stable trend in RMSD, showcasing an average deviation of approximately <3.5 Å (Singharoy et al., 2016; Zuo et al., 2017). Such profiles demonstrate the inherent stability of the system investigated herein.

However, exploring the RMSD of the hesperidin–KatG complex (Figure 7B) yielded intriguing findings, where the ligand had notable changes in RMSD throughout the simulation, thereby highlighting a significant orientation within the binding pocket of KatG. The hesperidin-bound complex (indicated in red) exhibited an initial increment in RMSD up to ∼0.6 Å during the early stage of the simulation and lasting until around 75 ns, with an unusual rise at around 60 ns. Thereafter, it gradually increased to 2.65 Å at 110 ns, which dipped to 2.1 Å at 120 ns before rising above 2.4 Å at 125 ns. A dip was then observed at around 130 ns to 1.8 Å, after which the curve remained stable till the end of the simulation. However, a notable observation in the hesperidin–MacB complex was the occurrence of fluctuations in the RMSD between 0 ns and 80 ns, characterized by few crests and troughs, indicating substantial conformational changes within the hesperidin-bound complex during this timeframe.

These fluctuations suggest the transient destabilization and restabilization of the complex, possibly due to dynamic interactions between hesperidin and the surrounding amino acid residues. Following this period of fluctuations, the RMSD stabilized at around 3.0 Å and remained relatively stable until the end of the 200 ns simulation. This remarkable stability highlights the robust and consistent natures of the complexes.

To investigate the flexibility and local changes in the secondary structural elements involved in hesperidin binding, the residual fluctuations of the three complexes were examined carefully by mainly focusing on the Cα motions (Figure 8). The Cα RMSFs of the three complexes displayed noteworthy fluctuations in the residues situated at the N-terminal and C-terminal regions, corroborating the findings obtained from the RMSD analysis (Figure 7). Interestingly, the ligand-bound structures exhibited lower fluctuations (Figure 8) at the points of contact (green lines) with the amino acid residues of the proteins, with an average RMSF of 2.4 Å for hesperidin–gidB and 2.0 Å for hesperidin–KatG even when the fluctuations around the residues between 210 ns and 220 ns had an RMSF value of above 4.0 Å.

In the hesperidin–MacB complex (Figure 8C), the fluctuations averaged between 2.5 Å and 3.0 Å around the points of contact of hesperidin and the amino acid residues of MacB. The observed changes in the Cα-RMSF values are probably a consequence of the stabilizing effects prompted by hesperidin binding to the protein’s flexible regions, emphasizing the inherent adaptability of the proteins (Dokainish et al., 2022; Ali et al., 2023). All three systems displayed steady RMSF trends with marginal distinctions, confirming cohesive behaviors under dissimilar conditions.

### 3.10 Protein–ligand contacts

**FIGURE 9:**
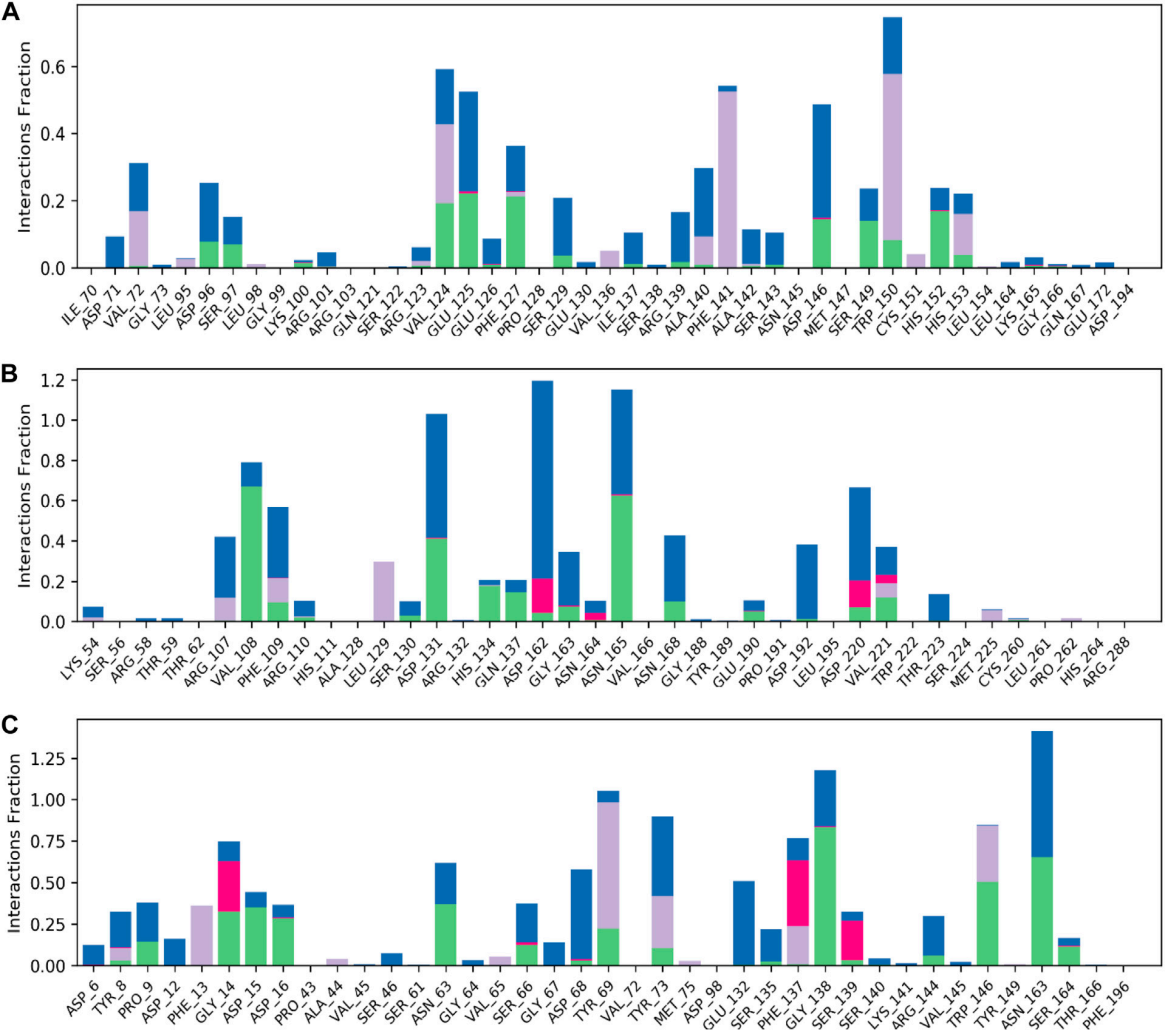
Normalized stacked bar charts representing the contact mappings of hesperidin with **(A)** gidB, **(B)** KatG, and **(C)** MacB. The green, gray, blue, and pink colors represent hydrogen bonds, hydrophobic interactions, water bridges, and ionic interactions, respectively.

**FIGURE 10:**
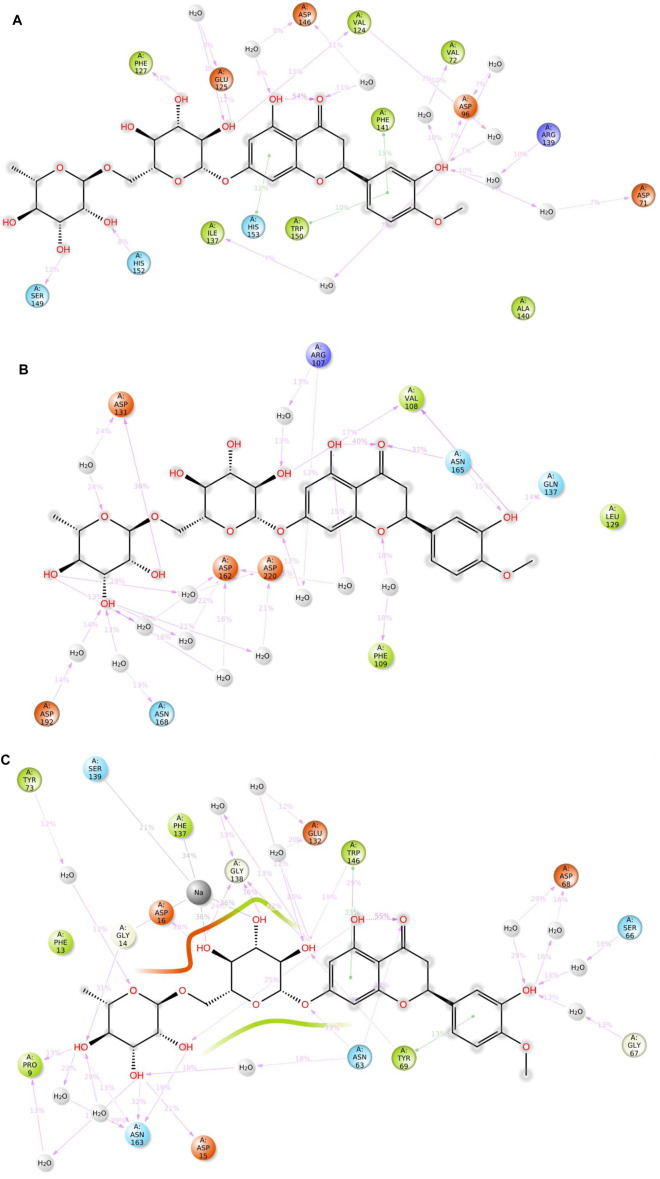
2D summary diagrams showcasing the preserved contacts from 200 ns of MD simulations between hesperidin and the *E. coli* proteins **(A)** gidB, **(B)** KatG, and **(C)** MacB.

The model trajectories were used to measure hesperidin’s binding affinity for identifying the stabilities of the three protein targets against it. The resulting structural chemistries witnessed during these modeling runs are depicted graphically in Figures 9, 10. These figures exemplify several interactions, such as hydrogen bonds in green, hydrophobic interactions in gray, ionic interactions in magenta, and water bridges in blue. The stacked bars (Figure 5) artfully represent the proportion of simulation time dedicated to maintaining each definite interaction.

For example, a value of 0.6 would indicate that 60% of the simulation time involved maintaining a particular interaction. In some cases, values exceeding 1.0 were acceptable, signifying the manifestation of multiple contacts between a ligand and the same protein subclass due to conformational changes in the protein structure. Furthermore, 2D interaction charts for the hesperidin and three target protein complexes are shown in Figure 10, furnishing insights into the preservation of contacts over the entire simulation trajectory.

It is noted that both the Vina-based docking (Figures 4–6) and MD simulations (Figures 9, 10) were able to uncover significant H-bond interactions between hesperidin and the active site residues of the three protein targets, which may likely indicate the correctness of compound docking within the binding pockets of the target proteins. This is an interesting outcome that hesperidin may likely be a good inhibitor of these proteins and consequently prevent the spread of AMR in *E. coli*. Post-simulation in hesperidin–gidB, most of the OH groups of hesperidin were found to form five water-mediated hydrogen bond contacts with six residues: Asp96, Asp146, Glu125, Val124, Val72, and Arg139. Interestingly, these interactions occurred in 7%–31% of the frames throughout the simulation trajectory. Furthermore, three residues were observed to contact hesperidin via hydrogen bonds without mediation by water: His152, Phe127, and Ser149. Finally, His153 and Trp150 showed pi-pi interactions with rings A and C of hesperidin (Figure 10).

As in gidB, the hesperidin-bound KatG had many H-bond formations during the docking event despite being ranked second in terms of affinity among the chosen natural compounds (Figure 4). However, the H-bond interactions were mostly maintained between 13% and 40% during the MD simulations (Figure 10B), which may be due to conformational changes in the protein and ligand [47–48]. Such molecular changes were attributed to the moderate fitness of hesperidin in the binding pockets. However, the complex exhibited six water-mediated interactions, all involving the beta-oriented OH: four with the positively charged Asp192, Asp131, Asp162, and Asp220 residues as well as two with Phe109 and Arg107. Some additional residues had interactions with the OH groups of hesperidin without water mediation, namely Gln137, Asn165, and Val108. These contacts were maintained between 13% and 40% of the MD timescale (Figure 10B), signifying their modest stabilities.

Finally, the hesperidin-bound MacB had few H-bond formations during the docking event despite being ranked best in terms of affinity among the chosen natural compounds (Figure 6). However, the H-bond interactions were mostly maintained between 12% and 36% during the MD simulations (Figure 10C), which may be attributed to positional variations in MacB and the ligand (Girdhar et al., 2019; Redij et al., 2019). Such molecular changes were ascribed to the reasonable fitness of hesperidin in the binding pockets. However, the complex exhibited ten water-mediated interactions, all involving the beta-oriented OH: three with the positively charged Asp16, Glu132, and Asp68 residues, with the remaining residues being Ser66, Gly67, Asn63, Asn163, Tyr73, Pro9, and Phe137. Some additional residues had interactions with the OH groups of hesperidin without water mediation, namely Asp15, Gly14, and Trp146. In addition, Trp146 and Tyr69 were observed to interact with rings A and C of hesperidin during the MD simulations (Figure 10C), signifying their modest stabilities as a result of the H bonds and pi-pi interactions.

### 3.11 PCA

**FIGURE 11:**
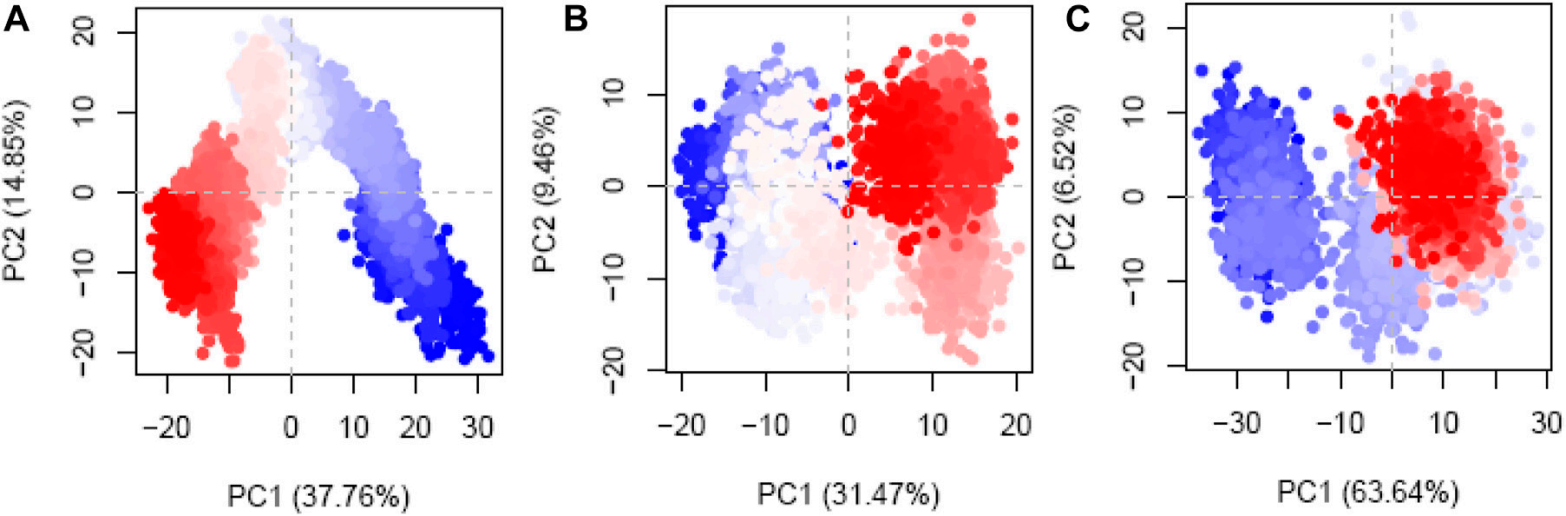
Principal component analysis (PCA) plots of the Cα atoms disclosing the first two eigenvectors in the conformational spaces of three different systems: **(A)** hesperidin–gidB, **(B)** hesperidin–KatG, and **(C)** hesperidin–MacB complexes.

PCA was implemented to meticulously measure the routes of the protein–ligand complexes during MD simulations. The goals of this inquiry were to attempt to make sense of the seemingly random, global atomic displacements observed within the amino acid residues (Hayward, 2023; Vuillemot et al., 2023). The protein’s layout includes stochasticity and globally uncorrelated motions, contributing to the diversity and adaptability of the trajectories (Alghamdi et al., 2023; Debnath et al., 2023). The covariance matrix records the movements of the internal coordinates over 200 ns, as projected onto a 3D space. With this information, orthogonal systems or eigenvectors can be used to better comprehend the predictable motions inside each trajectory. The Cα atoms of the hesperidin-bound gidB (Figure 11A) demonstrated a disordered configuration in the MD simulation trajectories associated with the PC1 and PC2 modes in the early and last frames.

In contrast, a more systematic arrangement emerged between the frames (highlighted in light to white), suggesting stable global correlated motions. In the case of KatG–hesperidin (Figure 11B), initial anisotropic motion was observed, which is attributed to the high mobility of the protein’s terminal regions in various directions; however, these motions became more ordered with time. Meanwhile, the Cα atoms of MacB linked to hesperidin (Figure 11C) exhibited more disordered configurations with moderate orientations in the PC1 and PC2 modes. This clustering of frames indicates cyclical motion, as shown in the MD trajectories, which results from the robust global conformational movements.

### 3.12 DCC analysis

To clarify the complex, symbiotic conformational dynamics within the three ligand-bound protein complexes expressed across spatially divergent protein domains in different systems, a meticulous and sophisticated DCC analysis was employed (Zhang et al., 2022; Zhu et al., 2022). It involved averaging over three replicates of precisely curated MD trajectories for each system. The cross-correlation (C*ij*) coefficients, which are a display of mathematical sophistication, gracefully traversed the entire range from −1 (purple) to 1 (blue), reflecting the exquisitely collinear correlation between the two Cα atoms (*i* and *j*). Here, positive coefficients (C*ij* > 0) indicate harmonious and synchronized movements of the two residues, whereas negative coefficients (C*ij* < 0) suggest that the two residues move in opposite directions. As the coefficients approach zero (C*ij* = 0), the correlated motions between the two residues reach a standstill, indicating no noticeable correlation. Throughout the analysis, the absolute values of C*ij* highlight the intensity of the correlated motions within the complexes, providing insights into the magnitudes of the molecular movements (Zhang et al., 2022; Zhu et al., 2022).

**FIGURE 12:**
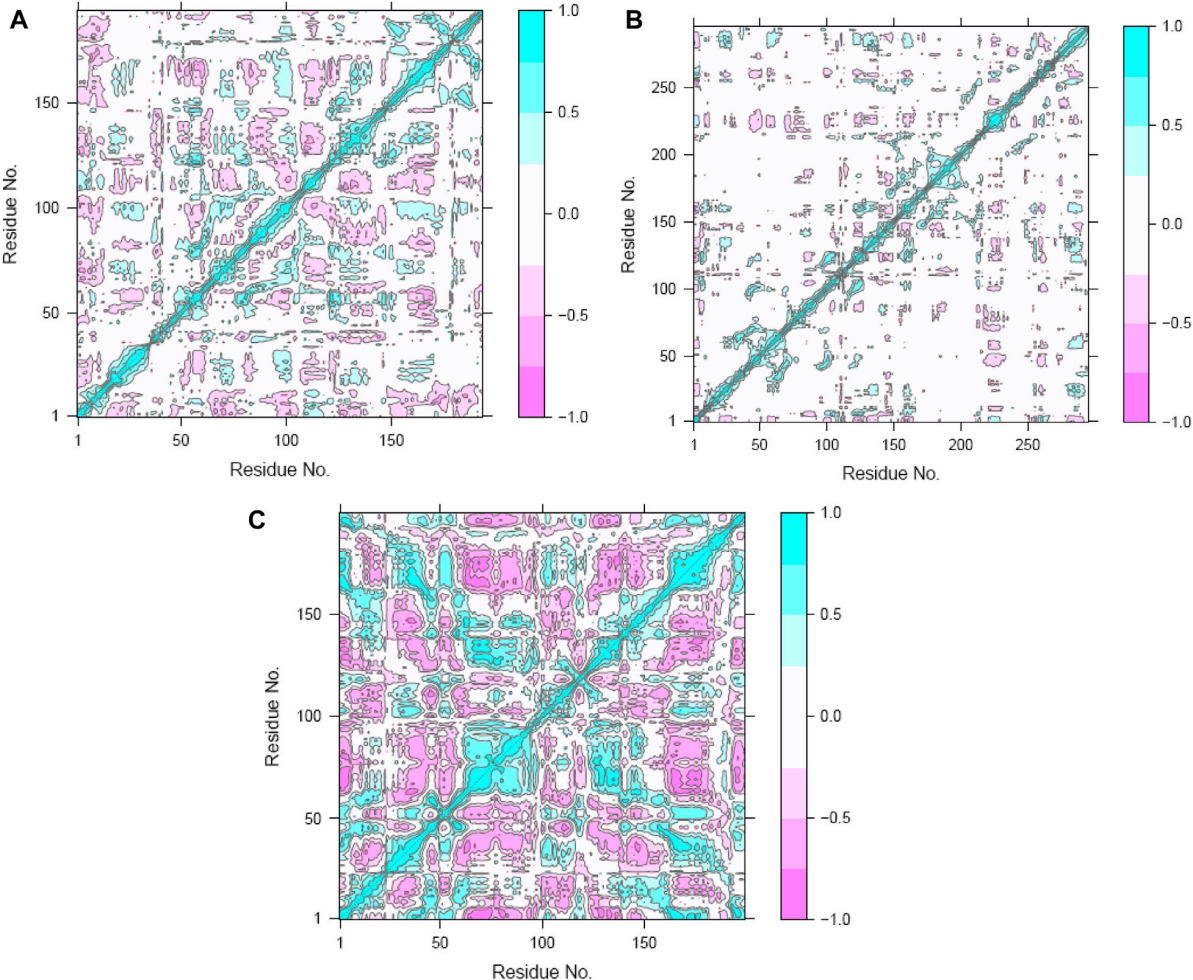
Dynamic cross-correlation maps (DCCMs) of the **(A)** hesperidin–gidB, **(B)** hesperidin–KatG, and **(C)** hesperidin–MacB complexes.

The C*ij* matrices, represented as two-by-two plots of the Cα C*ij* coefficients, revealed near-similar diagonal patterns of correlated and anti-correlated motions within the ligand-bound protein systems as well as interdomain motions between these systems (Figure 12A–C). Across all three systems, the intradomain movements of the three proteins showed enhanced correlated motions (visually depicted as diagonal blue lines), indicating concerted motions within these regions.

### 3.13 FEL investigation

**FIGURE 13:**
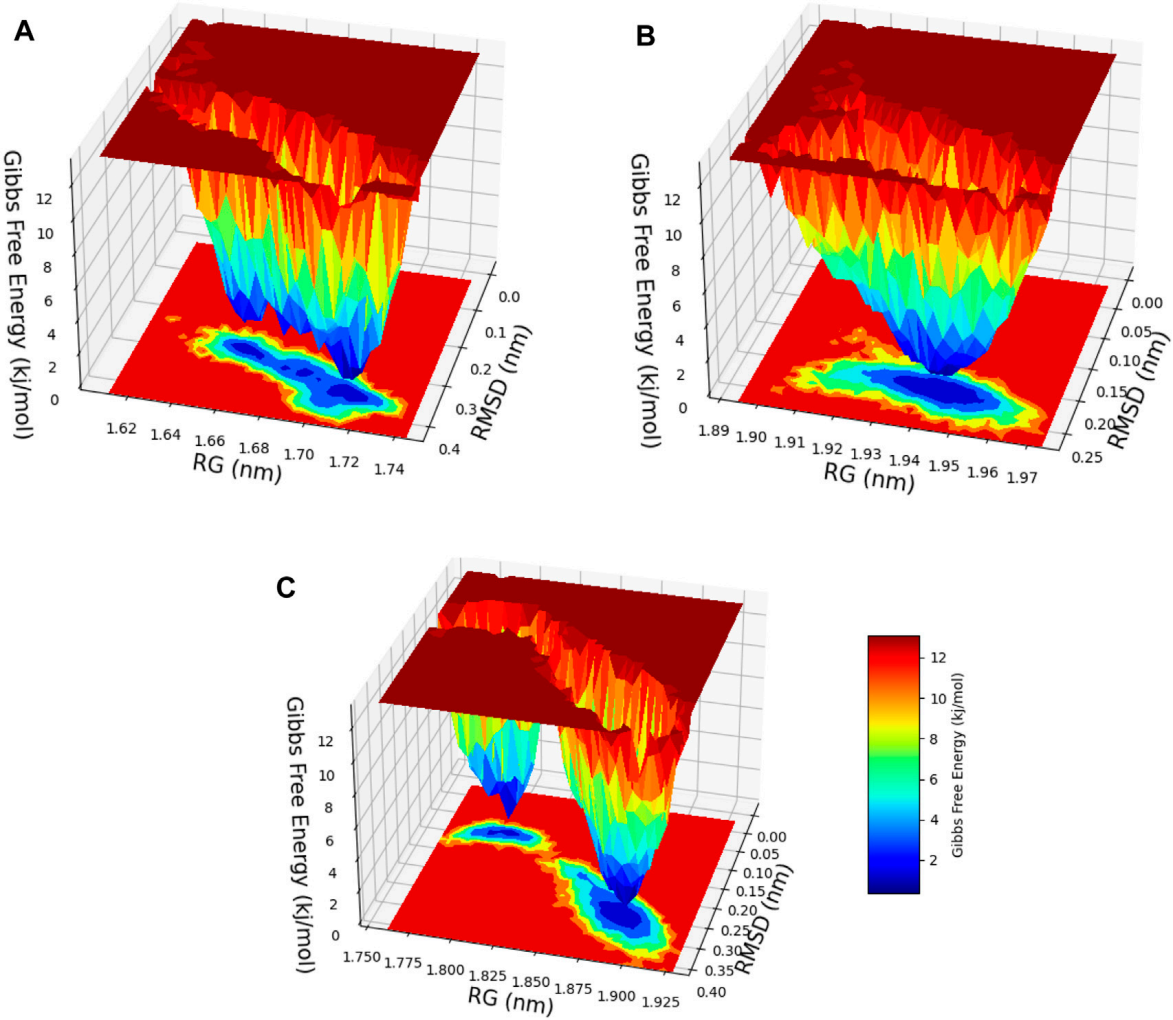
3D free-energy landscape (FEL) contour plots of the **(A)** hesperidin–gidB, **(B)** hesperidin–KatG, and **(C)** hesperidin–MacB clusters with respect to the RSMD and Rg.

After a meticulous appraisal of the configurational space explored via simulations, the FEL representation was constructed in contours. The FEL is a valuable model that involves mapping the free energy values to the configurational space, enabling identification of energetically favorable regions and conversion blockades to the system during its conformational changes or evolutions between different states (Fang et al., 2022). In this investigation, the FEL was calculated via RMSD and Rg values as the reaction coordinates for evaluating relevant clusters of the three systems of hesperidin (Figure 13). A noteworthy finding was the significant divergence in the free-energy profiles of the ligand-bound complexes (Figures 13A–C). The hesperidin-bound gidB showed one main energy basin with similar free-energy values of 0 kJ/mol and an extended spread along the Rg value from 1.64 nm to 1.74 nm (Figure 13A), whereas the hesperidin-bound KatG displayed only a single and sharp global energy minimum (Figure 13B).

The hesperidin-bound MacB showed two main energy basins (Figure 13C). Such deviations suggest that the binding of hesperidin induces noteworthy changes in the energetic landscapes of the three protein molecules, stabilizing them in preferred conformations during the interactions. The sizes and shapes of the minimal energy areas, represented by dark blue regions on the free-energy contour plots, provide crucial insights into the enhanced stabilities of the complexes. The presence of smaller, more centralized blue area(s) resembling funnel-like bottoms indicate more excellent stabilities of the corresponding complexes. This funnel-like shape suggests that the ligand-bound complex adopts a preferred conformation with minimal energy, making it highly stable and less susceptible to significant conformational changes (Sang et al., 2020; Baidya et al., 2022).

In principle, the binding with hesperidin stretches the protein and results in a change of the overall motions of the protein. Convincingly, applying PCA in the current work sheds light on the dynamic properties of the gidB, KatG, and MacB proteins as well as how they are influenced by ligand binding. The results suggest that hesperidin interactions are vital in shaping the conformational landscapes and firmness of the proteins, possibly impacting their functional behaviors in cellular processes.

Hesperidin behaviors noted in this work are not accidental as they have been reported to have significant bioactivities that can combat bacterial and other microorganisms to enhance immune functions (Alam et al., 2023; Rudrapal et al., 2023). Furthermore, hesperidin can disrupt the cell walls of bacteria and cause leakage of biological macromolecules, such as proteins and DNA, by generating reactive oxygen species (Bahador and Vaezi, 2023). Regarding antimicrobial activity, hesperidin has been known to exhibit robust effects against countless microorganisms, including bacteria, viruses, and fungi; its mechanisms of action involve the disruption of microbial cell membranes and inhibition of microbial enzymes (Akbari et al., 2023; Bahador and Vaezi, 2023; Hosawi, 2023). These findings suggest that hesperidin could be a potential natural antimicrobial agent for the prevention and treatment of microbial infections.

## 4 Conclusion

This study aimed to contribute to the ongoing quest for better therapeutics that can circumvent the AMR of *E. coli* by focusing on its MacB, gidB, and KatG proteins by identifying potential inhibitors from compounds attributed to plants with established antibacterial activities and utilizing a comprehensive approach involving resistance gene identification, molecular docking, MD simulations, and ADMET analysis. Remarkably, our findings reveal that hesperidin exhibits good binding affinities with MacB, KatG, and gidB, with BEs of −10.7 kcal/mol, −9.3 kcal/mol, and −6.7 kcal/mol, respectively, compared to their respective control drugs. Notably, hesperidin demonstrates plausible binding positions, good structural stability, and favorable pharmacokinetic profile. These findings suggest that hesperidin holds promise as a potential therapeutic agent for AMR associated with the MacB, gidB, and KatG genes of *E. coli*.

However, it is vital to consider the preliminary nature of these *in silico* findings, so further experimental studies are imperative for validating the efficacy of hesperidin as a viable treatment option for AMR associated with the MacB, gidB, and KatG genes of *E. coli*. These future investigations are necessary for valuable insights into bridging the gap between computational predictions and practical therapeutic applications, thereby paving the path for more effective and targeted treatments.

## Data Availability

The datasets presented in this study can be found in online repositories. The names of the repository/repositories and accession number(s) can be found in the article/Supplementary Material.
